# Supplementary material for: Outcomes in acute pulmonary embolism and their association with adherence to international recommendations around COVID-19 pandemic-induced hospital-strain: The experience in a Mexican tertiary care center
Source: PLoS One. 2026 Apr 29;21(4):e0347761. doi: 10.1371/journal.pone.0347761 (PMC13127952; doi:10.1371/journal.pone.0347761)
Supplement: S1 Fig — (DOCX) [file pone.0347761.s001.docx]

**S1 Fig. Flowchart of adherence criteria based on international recommendations for the management of acute pulmonary embolism.**


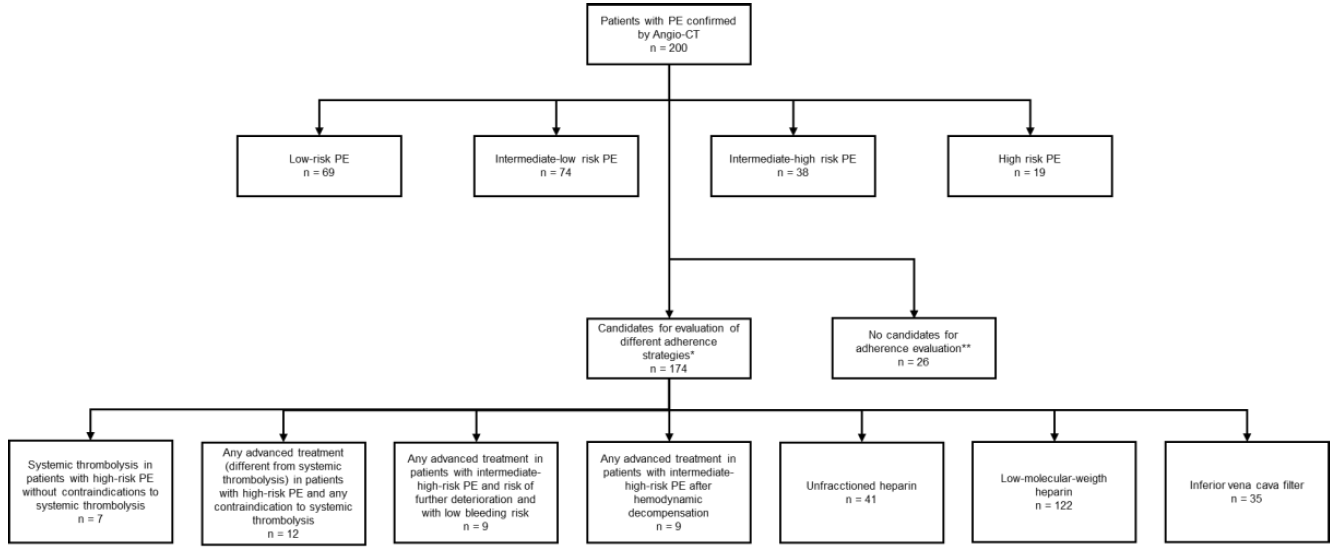


* Each of the patients was a candidate for evaluation of 1 or more of the criteria for adherence to clinical practice guidelines, so the sum of the participants by evaluation criteria does not add up to the total of the 174 patients initially considered to be evaluated. They were not considered candidates for evaluation of adherence to clinical practice guidelines due to 450 instances of incomplete information in the electronic clinical record regarding the treatment instituted.
