# Supplementary material for: Outcomes in acute pulmonary embolism and their association with adherence to international recommendations around COVID-19 pandemic-induced hospital-strain: The experience in a Mexican tertiary care center
Source: PLoS One. 2026 Apr 29;21(4):e0347761. doi: 10.1371/journal.pone.0347761 (PMC13127952; doi:10.1371/journal.pone.0347761)
Supplement: S1 Table — (DOCX) [file pone.0347761.s002.docx]

**S1 Table. Overall adherence to international recommendations by category.**

| **Non-adherence categories** | **COVID-19s**  **n (%)** | **Non-COVID-19s**  **n (%)** | **All population**  **n (%)** |
| --- | --- | --- | --- |
| Use of systemic thrombolysis in patients with high-risk PE without contraindications to systemic thrombolysis (n=7). | 2 (66.67) | 3 (75.00) | 5 (71.43) |
| Use of any advanced treatment (different from systemic thrombolysis) in patients with high-risk PE and any contraindication to systemic thrombolysis (n=12). | 5 (100.00) | 6 (85.71) | 11 (91.67) |
| Use of any advanced treatment in patients with intermediate-high-risk PE with risk of further deterioration and low bleeding risk (n=9). | 2 (100.00) | 6 (85.71) | 8 (88.89) |
| Use of any advanced treatment in patients with intermediate-high-risk PE after hemodynamic decompensation (n=9). | 0 (0.00) | 6 (75.00) | 6 (66.67) |
| Use of unfractionated heparin (n=41). | 9 (56.25) | 15 (60.00) | 24 (58.54) |
| Use of low-molecular-weight heparin (n=122). | 1 (2.27) | 9 (11.54) | 10 (8.20) |
| Use of inferior vena cava filter (n=35). | 16 (94.12) | 16 (88.89) | 32 (91.43) |

*Abbreviations: PE, pulmonary embolism; COVID-19s, COVID-19 season.*
