# Supplementary material for: Outcomes in acute pulmonary embolism and their association with adherence to international recommendations around COVID-19 pandemic-induced hospital-strain: The experience in a Mexican tertiary care center
Source: PLoS One. 2026 Apr 29;21(4):e0347761. doi: 10.1371/journal.pone.0347761 (PMC13127952; doi:10.1371/journal.pone.0347761)
Supplement: S2 Table — (DOCX) [file pone.0347761.s003.docx]

**S2 Table. Multivariate model for the composite outcome.**

| **Variable** | **Odds ratio** | **95% confidence interval** | ***P value*** |
| --- | --- | --- | --- |
| Age | 1.01 | 0.98–1.03 | 0.52 |
| Men | 1.86 | 0.95–3.64 | 0.07 |
| Year of diagnosis | 0.86 | 0.62–1.19 | 0.36 |
| COVID-19 season | 1.99 | 0.91–4.34 | 0.08 |
| Type of hospitalized patient | 1.06 | 0.39–2.84 | 0.92 |
| Charlson index | 0.99 | 0.80–1.21 | 0.89 |
| Presence of any chronic-degenerative comorbidity | 0.98 | 0.34–2.89 | 0.98 |
| Non-adherence | 2.36 | 1.23–4.54 | 0.01 |
